# A cost management model for hospital food and nutrition in a public hospital

**DOI:** 10.1186/s12913-014-0542-0

**Published:** 2014-11-13

**Authors:** Liliana Neriz, Alicia Núñez, Francisco Ramis

**Affiliations:** Department of Management Control and Information Systems, School of Economics and Business, Universidad de Chile, Santiago, Chile; Department of Industrial Engineering, Center of Advanced Studies in Process Simulation, Universidad del Bío Bío, Concepción, Chile

**Keywords:** Activity based costing, Diet costs, Nutrition costs, Activity based management for nutrition units

## Abstract

**Background:**

In Chile, the use of costing systems in the public sector is limited. The Ministry of Health requires hospitals to manage themselves with the aim of decentralizing health care services and increasing their quality. However, self-management with a lack of accounting information is almost impossible. On the other hand, nutrition department costs have barely been studied before, and there are no studies specifically for activity based costing (ABC) systems. ABC focuses on the process and traces health care activities to gain a more accurate measurement of the object costs and the financial performance of an organization.

**Method:**

This paper uses ABC in a nutrition unit of a public hospital of high complexity to determine costs associated with the different meals for inpatients. The paper also provides an activity based management (ABM) analysis for this unit.

**Results:**

The results show positive effects on the reduction of costs for the nutrition department after implementing ABC/ABM. Therefore, there are opportunities to improve the profitability of the area and the results could also be replicated to other areas in the hospital. ABC shed light on the amount of nutritionist time devoted to completing paperwork, and as a result, system changes were introduced to reduce this burden and allow them to focus on more relevant activities. Additional efficiencies were achieved through the elimination of non-value adding activities and automation of reports. ABC reduced the cost of the nutrition department and could produce similar results in other areas of the hospital.

**Conclusions:**

This is a practical application of a financial management tool, ABC, which would be useful for hospital managers to reduce costs and improve the management of the unit. This paper takes ABC and examines its use in an area, which has had little exposure to the benefits of this tool.

## Background

Improving nutritional status in any country is a challenge. It has been well documented that poor people are at increased risk for specific health conditions and diseases given their financial situation, lack of education, poor nutrition and health status [1]. In this way, nutrition is a factor in exacerbating inequalities in health. Hospitals also have an important nutritional role in preventing illness and maintaining the health of their patients; this produces a constant need to improve their efficiency and productivity. However, achieving hospital efficiency is not easy, particularly nowadays when there are many hospitals suffering from the absence of administrative and financial autonomy, and also have budgets that ignore the actual services provided by them [2].

Thus, nutrition is an important determinant of health for any patient. Adequate patient meals are an essential part of hospital treatment and the consumption of a balanced diet is crucial for a patient’s recovery. Although diet is just one of the lifestyle factors that influences quality of life, a proper diet combined with aftercare and nutritional education may influence the quality of the patient’s future health and life. The importance of hospital food and its benefits have been well studied [3,4]. However, the provision of hospital meals is a difficult process aggravated by the potential of the patient’s malnutrition [5,6].

People tend to forget the importance of hospital food services when comparing other clinical activities, and meal services are more prone to be subject to a budgetary cut than other services [7]. Therefore, it is difficult to find the balance between delivering quality food services and appropriate costs, mainly because of the lack of competencies required to perform this task and tools to enable proper management of the services. In addition, the quality of hospital food services has a critical effect on patient satisfaction [8], which influences the patient’s perception of the quality of the services provided by the hospital. The potential impact on both health status and patient satisfaction emphasizes the need to achieve quality in the food and nutritional services provided, which is not independent of the decision of how to allocate limited resources.

In any hospital it is a challenge to control health care expenses. In fact, escalating health care costs due to changes in the age distribution of the population increases in the levels of expectation for health care services, and the application of new technologies for health care delivery urge governments towards cost containment solutions. As a result, there is a need for more accurate data on health care services costs, which is useful for policy making as well as internal management decisions [9-11].

In view of producing more accurate cost estimates, health care organizations have started to invest in sophisticated management tools, including costing systems. ABC is a cost accounting system, which: (i) allows cost efficiency without negative impact on the quality of services, (ii) provides information for management, and (iii) and aids with continuous quality improvements [12]. ABC allocates indirect costs to services using a multistep allocation procedure on the basis of activity consumption. Based on the information provided by ABC, there is a set of actions and improvements that can be performed in a process to satisfy customers and reduce or control costs, known as ABM [13].

In Chile, there is a lack of health care cost accounting systems for decision-making processes in public hospitals, including nutrition units. There are many benefits associated with the use of more sophisticated techniques, for example, Álvarez et al. have shown that improving the quality of information systems in clinical nutrition will have a positive impact on the overall results of the hospital when measured in terms of effectiveness, efficacy or quality [14].

The aim of this study is to describe the development and application of ABC and ABM systems for a nutrition department in a hospital of high complexity.

### Activity based costing

ABC is an information system that not only maintains and processes data on activities and services to allocate costs, but also supports management decision-making. ABM describes the decision-making process that uses the information provided by ABC to comply with the objectives of any organization. According to Kaplan and Cooper [13], ABC helps managers by: defining prices of products/services, reducing costs by improving processes, focusing on quality and security, eliminating activities that do not add value and performing benchmarking.

ABC focuses on the processes and activities that take place in an organization. Indirect costs are accumulated for each activity as a separate cost object and then applied to products or services. There is extensive literature about studies and applications of ABC in North America and Europe, but not in Latin America. To mention a few: Barros et al. [15], Ross [16], Udpa [17], Laurila et al. [18], Roybal et al. [19], Dodson et al. [20], Canby [21] and Car [22]. All these authors present evidence of benefits associated with the implementation of ABM in health care organizations.

For nutrition units, ABC can play an essential role in maximizing reimbursement revenues and lowering operational costs without compromising the quality of the services. The importance of delivering high-quality nutritional care for patients at risk of malnutrition and its effects on clinical outcomes and costs savings has been well documented by Smith and Smith [23]. There are many studies that present experiences in nutrition units using cost-benefit and cost-effectiveness analysis such as Hedberg et al. [24], Brugler and Berstain [25], among others.

A paper presented by Pereira [26] examines cost-management methods in a Brazilian nutrition and diet unit. The authors compare the weight calculation costing method with the absorption costing method showing great discrepancies between these two methodologies. The authors suggest the adoption of more accurate methodologies to assign costs, such as ABC. Despite Pereira’s work, ABC/ABM for nutrition units has not been widely discussed in the literature.

## Methods

ABC involves a two-step process: the first step traces resources costs to activities, followed by a second step that traces activities costs to products or services to determine their cost. This paper adopts the methodology suggested by Kaplan and Cooper [13], which has been previously implemented by Canby [21] and Roybal et al. [19], to name a couple. The methodology consists of: first, thoroughly analyze the organization processes; second, identify and classify activities associated to the processes; third, identify resource drivers to assign indirect costs to activities; and fourth, assign activity costs to cost objects through the use of activity drivers.

### Organization process analysis

To understand the processes involved in the nutrition department, three information sources were considered: interviews with key personnel, observation of the different tasks performed in the unit, and measurement of process time.

### Activity analysis

Once the different processes are recognized, activity analysis within ABC evaluates resource consumption though the identification of activities. Activity analysis provides information such as what task is done, how it is done, and time necessary to perform the task. Activity data was collected through observation and interviews, and gathering data from existing documents and records. As a result, 24 nutritional activities were identified in five major areas.

### Cost drivers

Resource drivers and activity drivers were collected based on cause-and-effect relationships between activities and resources, and between activities and cost objects, respectively.

### Cost objects

Cost objects for the nutrition unit were understood as the result of the production process with an economic sense. In this study the cost objects correspond to the different clinician diets that are recommended for each patient. In this study 36 types of diets are provided by the hospital.

**Figure 1:**
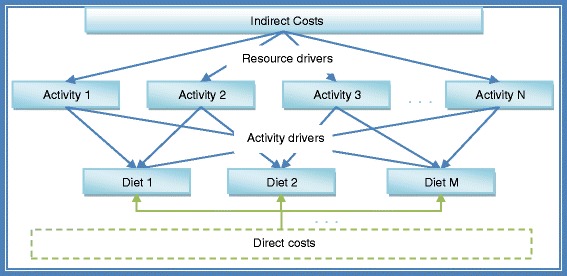
**ABC methodology.**

In summary, ABC uses resource drivers to assign indirect costs to activities, and then the costs of activities are assigned to cost objects (diets) based on the appropriate activity driver. See Figure 1.

### Data sources

ABC methodology was applied and information was obtained from the Guillermo Grant Benavente Hospital in Concepción, Chile, which is a hospital of high complexity, for all ages, with a capacity of 927 beds. The data presented in the ABC model is from January-December 2005 when the model was first implemented. During 2005, the hospital had a total of 40,466 patient discharges, an average of 725 hospitalized patients per day, and approximately 2,260 meals were prepared per day. This model has been updated in the hospital to date.

### Ethical approval and data protection

For the course of this study a confidential agreement was settled between the University of Chile, the Guillermo Grant Benavente Hospital, and FONDEF. The study design was approved by the ethics committee from the Faculty of Economics and Business of the University of Chile.

### Consent

Consent from the patient was not required, as this study reports an assessment of the hospital costs not at the patient level.

## Results

This section details the development and implementation of ABC in the nutrition department of a hospital of high complexity in Chile. A key for this case study was the willingness of the nutrition unit to provide access to all relevant information. We have divided this section into four steps: in the first step, we explain the processes involved in the nutrition department; second, we present an activity analysis for each relevant activity performed by the nutrition unit. In a third step, we calculate the cost of the different activities; and fourth, we calculate the cost of the different diets.

### Step 1: process analysis

Process analysis provides basic information about the activities performed in the nutrition department. The main processes identified in the department are: nutritional care, planning and food production.

- Nutritional care: This process starts with the visit to the patient to identify the appropriate diet in accordance with the physician’s specifications, and ends with a daily diet report specifying the number of different meals for the day. Six nutritionists, one per floor, participate in this process, which includes two visits to each patient per day.
- Planning: The planning process uses the daily diet report to calculate the ingredients required for food production and its availability for each meal. Four nutritionists and one assistant participate in this process.
- Food production: this process includes activities that range from receiving food supplies to preparing the food, to hygiene. 24 kitchen assistants and 3 nutritionists participate in this process.

### Step 2: activity analysis

An activity dictionary was constructed for a typical nutrition unit based on: the process analysis, several interviews, direct observation and time measurements. We identified 24 activities classified into 5 categories: (i) nutritional care activities, (ii) administrative activities, (iii) food production activities, (iv) supervision, (v) distribution and hygiene activities. Table 1 shows the activity dictionary.

**Table 1:** **Activity dictionary for the nutrition department**

**N°**	**Activity**	**Description**	**Type of activity**
1	Visit patients	Nutritionist visits the patient to assign daily diet.	Nutritional care
2	Count diets	Nutritionist counts the different diets.	Planning
3	Create report	Nutritionist prepares a report with the diets for the day.	Planning
4	Compute ingredients	Nutritionist counts the amount of ingredients required to order them from storage and different suppliers.	Planning
5	Request ingredients	Nutritionist requests the ingredients from storage or calls suppliers to check availability.	Planning
6	Receive ingredients	Assistants receive ingredients to control for quality and quantity, and then store the food.	Food Production
7	Control reception of ingredients	Nutritionist verifies that the reception of ingredients is appropriate according to the defined standards.	Food Production
8	Prepare desserts and salads	Kitchen assistants prepare desserts and salads for the day or next day accordingly.	Food Production
9	Control desserts and salad	Nutritionist supervise that the preparation of dessert and salads is performed correctly and meets the standards set by the hospital in terms of safety and hygiene.	Food Production
10	Prepare ingredients	Kitchen assistants wash, peel, chop, and disinfect food.	Food Production
11	Control preliminary preparation of ingredients	Nutritionist supervise that the preliminary preparation of the food is performed correctly and meets the standards set by the hospital in terms of safety and hygiene.	Food Production
12	Prepare Food	Kitchen assistants cook the food for the different meals.	Food production
13	Control food preparation	Nutritionist supervise that the food is prepared correctly and meets the standards set by the hospital in terms of safety and hygiene.	Food Production
14	Prepare trays	Kitchen assistants prepare patients’ trays for the different meals.	Food production
15	Control preparation of the trays	Nutritionist reads the daily diet report to load the trays accordingly.	Food Production
16	Distribute to patients	Assistants distribute the food to the patients.	Food Production
17	Nutritional control	Nutritionists verify that the diet plan correspond to the patient.	Nutritional care
18	Remove trays	Assistants remove trays from the patients and take them back to the kitchen.	Food Production
19	Wash trays	Kitchen assistants wash and put away the trays according to the standards set by the hospital in terms of safety and hygiene.	Food Production
20	Wash cooking implements	Kitchen assistants wash and put away cooking implements such as containers, pots, pans, etc. according to the standards set by the hospital in terms of safety and hygiene.	Food Production
21	Wash control	Nutritionist supervises that the washing and putting away activities of the trays and cooking implements is performed correctly and meets the standards set by the hospital in terms of safety and hygiene.	Food Production
22	Clean area	Assistant cleans the kitchen and office area according to the standards set by the hospital in terms of safety and hygiene.	Food Production
23	Remove garbage	Assistant removes garbage.	Food Production
24	Coordinate nutrition department	General coordination activities that involve organization of shifts, budgetary control, alignment with other areas, etc.	Planning

Source: Own elaboration.

We removed some activities from the original activity dictionary, which includes activities related to the provision of food for the workers of the hospital because they are not the core of the hospital and the subject matter for this study.

### Step 3: activity costs

In order to determine the costs of the 24 activities previously identified, and following the ABC model illustrated in Figure 1, we assigned the cost of the resources to the activities using resource drivers. Table 2 shows indirect costs and resource drivers.

**Table 2:** **Indirect costs and resource drivers**

**N°**	**Resources**	**Resource driver**	**Cost (US$ per month)**
1	Nutritionists salaries	Labor hours	$ 101,188
2	Clinical nutritionists salaries	Percentage of use	$ 125,516
3	Assistants salaries	Percentage of use	$ 295,205
4	Secretary salary	Square meters	$ 5,303
5	Cleaning supplies	Square meters	$ 25,032
6	Library supplies	Square meters	$ 1,119
7	Water	Square meters	$ 7,952
8	Steam boiler	Minutes of use	$ 40,781
9	Gas	Activity duration	$ 810
10	Electricity	Activity duration	$ 13,173
11	Telephone	Activity duration	$ 1,027
12	Equipment depreciation	Depreciation	$ 22,975
13	Furniture depreciation	Activity duration	$ 3,195
14	Rooms depreciation	Activity duration	$ 11,524
15	Equipment maintenance	Activity duration	$ 6,713
16	Garbage	Kilos	$ 6,573

Note: The Chilean peso is the currency in Chile. Conversion rate: CLP/USD = $ 502 (January 2014).

Source: Own elaboration.

We use equation 1 to calculate activity costs:

1 $$ \begin{array}{cc}\hfill \mathrm{Activity}\ \mathrm{cost}={\displaystyle {\sum}_{i=1}^n\%\  Resourc{e}_i\times Resourc{e}_i Cost;}\hfill & \hfill i=1\dots n\  resources\hfill \end{array} $$

Equation 1 shows that activity costs correspond to the proportion of resources used by each activity multiplied by the cost of the resources. Table 3 presents activity costs for our case study.

**Table 3:** **Activity costs for the nutrition department**

**N°**	**Activity**	**Cost (US$ per month)**
1	Visit patients	$ 60,780
2	Count diets	$ 26,154
3	Create report	$ 4,459
4	Compute ingredients	$ 15,730
5	Request ingredients	$ 1,559
6	Receive ingredients	$ 40,697
7	Control reception of ingredients	$ 12,146
8	Prepare desserts and salads	$ 30,849
9	Control desserts and salad	$ 13,348
10	Prepare ingredients	$ 46,375
11	Control preliminary preparation of ingredients	$ 4,207
12	Prepare Food	$ 64,750
13	Control food preparation	$ 4,302
14	Prepare trays	$ 43,179
15	Control preparation of the trays	$ 16,223
16	Distribute to patients	$ 31,834
17	Nutritional control	$ 19,798
18	Remove trays	$ 21,367
19	Wash trays	$ 58,503
20	Wash cooking implements	$ 29,409
21	Wash control	$ 4,211
22	Clean area	$ 7,911
23	Remove garbage	$ 797
24	Coordinate nutrition department	$ 22,303

Note: The Chilean peso is the currency in Chile. Conversion rate: CLP/USD = $ 502 (January 2014).

Source: Own elaboration.

The difference between the total indirect cost and the total activity cost comes from eliminating the activities of feeding hospital employees.

### Step 4: diet costs

An inpatient receives four meals during the day: breakfast, lunch, snack break, and dinner. Some of the most recurrent diets in the hospital are: liquid diet, porridge diet, soft-food diet, no-residue soft diet, low-fat diet, and full diet. This hospital elaborates 36 different diets that we have organized into 16 different groups [27].

- Group 1: common, no-restriction, full diet.
- Group 2: Soft-food diet, no-salt soft-food diet, low-fat diet.
- Group 3: Low-potassium soft-food diet, no-salt low-potassium soft-food diet.
- Group 4: No-residue soft diet.
- Group 5: Diabetic diet, no-salt diabetic diet, Giovanetti diabetic diet.
- Group 6: Low potassium no-salt diabetic diet.
- Group 7: No-residue diabetic diet, Low-potassium Giovanetti diet, No-salt low-potassium Giovanetti diet.
- Group 8: Carbohydrate diet, carbohydrate no-salt diet, carbohydrate porridge, Carbohydrate no-salt porridge.
- Group 9: Porridge, no-salt porridge, no-residue porridge, no-salt no-residue porridge, semi-liquid porridge.
- Group 10: Diabetic porridge, no-salt diabetic porridge.
- Group 11: No-residue diabetic porridge.
- Group 12: Giovaneti diet, No-salt Giovaneti diet, Giovanetti porridge, No-salt Giovanetti porridge.
- Group 13: Low-calorie diet.
- Group 14: Liquid, no-salt liquid.
- Group 15: Pension.
- Group 16: Adult patient breakfast, pediatric patient breakfast

The consumers of these diets correspond to patients hospitalized in the clinical services at the hospital. Therefore, the meals are carefully prepared, cover the nutritional needs of the patient, are technically planned diets and are elaborated with optimal sanitation standards of the facilities. As previously mentioned, a typical patient diet includes four meals (breakfast, lunch, snack break, and dinner), and can sometimes include additional snacks between the main dishes. In summary, the cost objects include:

- Lunch and dinner: They can vary between the groups 1 to 15.
- Breakfast and snack break: group 16.

To determine the costs of the groups of meals, and according to the ABC model illustrated in Figure 1, we assigned activity costs to the group of diets using cost drivers. The cost drivers were chosen based on their causal relationship with the product, certainty and accuracy to the respective assigned costs. Table 4 shows activities, and cost drivers.

**Table 4:** **Activity costs and costs drivers**

**N°**	**Activity**	**Cost drivers**
1	Visit patients	Seconds per patient
2	Count diets	Seconds per patient
3	Create report	Labor hours
4	Compute ingredients	Minutes per diet
5	Request ingredients	Labor hours
6	Receive ingredients	Number of meals
7	Control reception of ingredients	Labor hours
8	Prepare desserts and salads	Number of meals
9	Control desserts and salad	Labor hours
10	Prepare ingredients	Number of preparations
11	Control preliminary preparation of ingredients	Labor hours
12	Prepare Food	Minutes of food preparation
13	Control food preparation	Labor hours
14	Prepare trays	Number of meals
15	Control preparation of the trays	Labor hours
16	Distribute to patients	Seconds per patient
17	Nutritional control	Labor hours
18	Remove trays	Number of patients
19	Wash trays	Number of trays
20	Wash cooking implements	Number of food preparations
21	Wash control	Labor hours
22	Clean area	Labor hours
23	Remove garbage	Number of food preparation
24	Coordinate nutrition department	Labor hours

Source: Own elaboration.

We use equation 2 to assign the costs of the activities to the different group of diets:

2 $$ \begin{array}{cc}\hfill \mathrm{Diet}\ \mathrm{cost}={\displaystyle {\sum}_{i=1}^n\%\  Activit{y}_i\times Activit{y}_i Cost;}\hfill & \hfill i=1\dots n\  activities\hfill \end{array} $$

Table 5 presents the indirect costs of the groups of diets for our case study.

**Table 5:** **Diet costs**

**Activity**	**Diet**	**Indirect Costs (US$)**	**Number of meals**	**Indirect unit costs (US$ per month)**
Group 1	Full diet	$ 20,520	23,708	0.87
Group 2	Soft-food diet, no-salt soft-food diet, low-fat diet	$ 184,032	212,630	0.87
Group 3	Low-potassium soft-food diet, no-salt low-potassium soft-food diet	$ 1,495	1,790	0.83
Group 4	No-residue soft diet	$ 6,227	7,456	0.84
Group 5	Diabetic diet, no-salt diabetic diet, Giovanetti diabetic diet	$ 40,964	47,866	0.86
Group 6	Low potassium no-salt diabetic diet	$ 624	746	0.84
Group 7	No-residue diabetic diet, Low-potassium Giovanetti diet, No-salt low-potassium Giovanetti diet	$ 1,370	1,642	0.83
Group 8	Carbohydrate diet, carbohydrate no-salt diet, carbohydrate porridge, Carbohydrate no-salt porridge	$ 2,390	2,834	0.84
Group 9	Porridge, no-salt porridge, no-residue porridge, no-salt no-residue porridge, semi-liquid porridge	$ 43,157	51,592	0.84
Group 10	Diabetic porridge, no-salt diabetic porridge	$ 9,839	11,780	0.84
Group 11	No-residue diabetic porridge	$ 383	448	0.85
Group 12	Giovaneti diet, No-salt Giovaneti diet, Giovanetti porridge, No-salt Giovanetti porridge	$ 1,744	2,090	0.83
Group 13	Low-calorie diet	$ 374	448	0.84
Group 14	Liquid, no-salt liquid	$ 25,140	29,376	0.86
Group 15	Pension	$ 6,361	7,604	0.84
Group 16	Group 16: Adult patient breakfast, pediatric patient breakfast	$ 57,788	745,920	0.08

Note: The Chilean peso is the currency in Chile. Conversion rate: CLP/USD = $ 502 (January 2014).

Source: Own elaboration.

Table 5 shows that the number of lunches and dinners corresponds to 402,010 annual meals, and the number of breakfasts and snacks equal 745,920 meals. This difference occurs for several reasons; one of them is that on average there were 725 hospitalized patients per day, of which 193 were discharged during the day, most of the time after they have their breakfast. Also, the hospital sometimes includes additional snacks between the main dishes. And a final reason relates with the time of patient procedures and the next meal they can ingest; procedures are usually performed during the day, so patients just eat a snack or dinner later or just have breakfast the next day.

**Figure 2:**
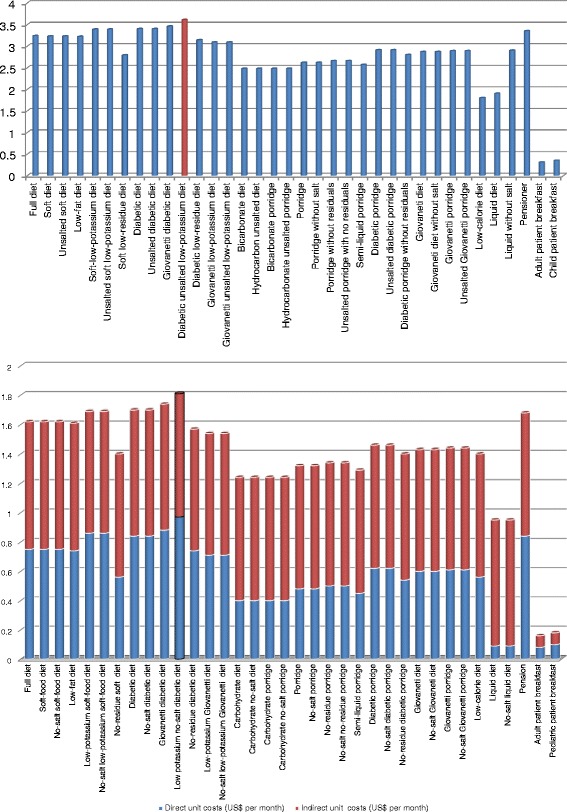
**Total unit costs per diet.**

The last step to obtain the total cost per diet was to add the indirect costs with the direct costs, which are the ingredients that we are able to identify directly with the diet (examples: potatoes, carrots, etc.). The direct costs were computed for each diet; these results are shown in Table 6, and summarized in Figure 2.

**Table 6:** **Total cost by diet**

**Diet**	**Group**	**Number of meals**	**Direct unit costs (US$ per month)**	**Indirect unit costs (US$ per month)**	**Total unit cost (US$ per month)**
Full diet	1	23,708	0.75	0.87	1.62
Soft-food diet	2	141,952	0.75	0.87	1.61
No-salt soft-food diet	2	54,872	0.75	0.87	1.61
Low-fat diet	2	15,806	0.74	0.87	1.61
Low-potassium soft-food diet	3	1,342	0.86	0.83	1.69
No-salt low-potassium soft-food diet	3	448	0.86	0.83	1.69
No-residue soft diet	4	7,456	0.56	0.84	1.40
Diabetic diet	5	16,850	0.84	0.86	1.70
No-salt diabetic diet	5	30,270	0.84	0.86	1.70
Giovanetti diabetic diet	5	746	0.88	0.86	1.73
Low potassium no-salt diabetic diet	6	746	0.97	0.84	1.81
No-residue diabetic diet	7	746	0.74	0.83	1.57
Low-potassium Giovanetti diet	7	448	0.71	0.83	1.54
No-salt low-potassium Giovanetti diet	7	448	0.71	0.83	1.54
Carbohydrate diet	8	1,492	0.40	0.84	1.24
Carbohydrate no-salt diet	8	596	0.40	0.84	1.24
Carbohydrate porridge	8	298	0.40	0.84	1.24
Carbohydrate no-salt porridge	8	448	0.40	0.84	1.24
Porridge	9	41,452	0.48	0.84	1.31
No-salt porridge	9	8,946	0.48	0.84	1.31
No-residue porridge	9	448	0.50	0.84	1.33
No-salt no-residue porridge	9	448	0.50	0.84	1.33
Semi-liquid porridge	9	298	0.45	0.84	1.28
Diabetic porridge	10	6,412	0.62	0.84	1.46
No-salt diabetic porridge	10	5,368	0.62	0.84	1.46
No-residue diabetic porridge	11	448	0.54	0.85	1.39
Giovaneti diet	12	448	0.60	0.83	1.43
No-salt Giovaneti diet	12	746	0.60	0.83	1.43
Giovanetti porridge	12	448	0.61	0.83	1.44
No-salt Giovanetti porridge	12	448	0.61	0.83	1.44
Low-calorie diet	13	448	0.56	0.84	1.40
Liquid diet	14	28,928	0.09	0.86	0.95
No-salt liquid diet	14	448	0.09	0.86	0.95
Pension	15	7,604	0.84	0.84	1.67
Adult patient breakfast	16	647,476	0.08	0.08	0.15
Pediatric patient breakfast	16	98,444	0.10	0.08	0.18

Note: The Chilean peso is the currency in Chile. Conversion rate: CLP/USD = $ 502 (January 2014).

Source: Own elaboration.

Figure 2 shows the most expensive diet is the Diabetic diet. The elevated costs are due to the high costs of the ingredients compared to the other diets.

The information provided by the ABC system allows us to know the cost of food for a typical inpatient in the hospital. Let’s assume that we have an adult patient who has four meals and was diagnosed with a soft-food diet; the cost per day for this patient is US$3.52, which is detailed as follows:

- Breakfast $0.15
- Lunch $1.61
- Snack break $0.15
- Dinner $1.61
- Total cost per day $3.52

Similar analyses can be conducted for all the diets and different patients. Therefore, ABC can be helpful with calculation of actual unit cost of a patient meal. All this information is now the cornerstone for the ABM system. An ABM system for this nutrition unit will be based on feedback from the management of the unit and the results from the accounting system. Management needs to analyze the results obtained to identify the unit’s deficiencies with the aim of improving the activities that are carried out in the unit and reducing costs.

## Discussion

**Figure 3:**
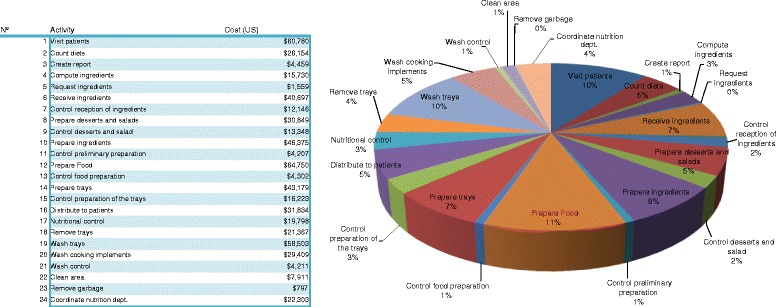
**Percentage of activity costs.**

The results provide insight into the actual costs of the patients’ meals for the hospital, but they also provide valuable information about the activities carried out in the hospital. Figure 3 presents the activity costs for the whole nutrition unit. It can be appreciated that the most expensive activities are preparing food (11%), washing and putting away trays (10%), and visiting the patients (10%).

**Figure 4:**
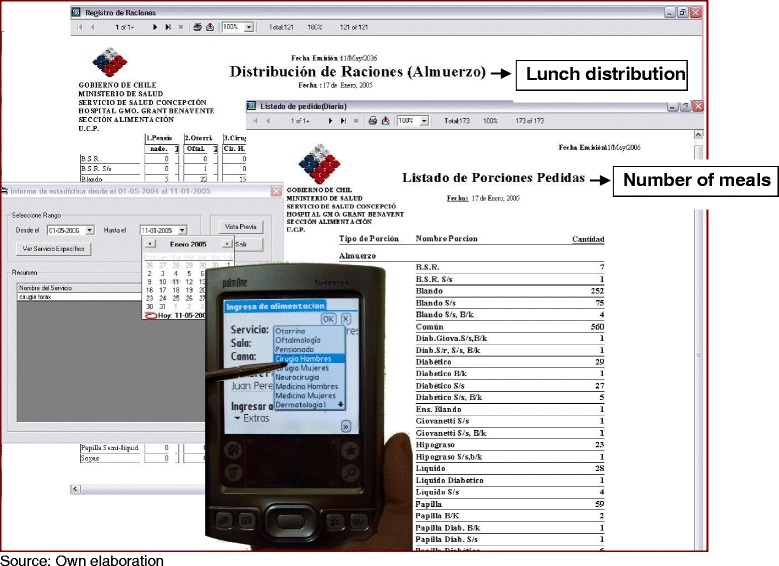
**Electronic system implemented in the nutrition unit.**

This information provides nutritionists and administrators with a common language with which to discuss the link between costs and procedures, and also provides alternatives in case reduction of costs or activity improvement is required. At this point, let us provide an example of how ABM was applied for the “Visit Patients” activity implemented in this hospital, and accounted for 10% of the total costs. The nutritionists were in charge of performing this activity daily, visiting inpatients and evaluating their diets twice a day. The main activities of the clinical nutritionist should be oriented to the assessment and care of the patients, but those activities were displaced for administrative activities, which accounted for over 60% of the cost in the wages of the professionals. Nutritionists were asked to fill out several forms indicating the patients’ meals. Each nutritionist prepared a form by hand, and after they finished visiting the patients, a larger form compiling all this information was also prepared by hand. Thus, an important part of the nutritionists’ day was consumed by filling out paperwork. The administration took into consideration the costs of the activities, and using a more managerial approach, came up with the solution of implementing technological tools that help nutritionists to register the diets of the different patients and avoid the use of all the paperwork (a photo of the new system is presented in Figure 4). As a result, a scarce nutritional resource was liberated to perform more relevant activities, by approximately one hour a day. The hour freed up by the nutritionists is now devoted to educating patients regarding their future diets and their impact on patient recovery and health status. Also, nutritionists now have more time to investigate new balanced diets for patients. All these activities are more relevant from a point of view of patient relapse and prevention of future diseases. Non-value-adding activities were also eliminated, such as counting diets and creating reports, as the reports are now automatically created. Finally, this leads to a reduction of costs by eliminating low-value high-cost activities and continued improvement of the unit. It is expected that ABC could produce similar results in other areas of the hospital.

## Conclusion

Many ABC models for health care institutions exist in the literature; in surgery departments [28], nursing administration [29], radiology department [18,30], nuclear medicine [31], prenatal care [32], renal dialysis clinic [33] and intensive care unit [34], among others. The cost objects may be a disease, a homogeneous group of patients, a medical procedure, etc. However, there are no costing models for a nutrition unit. The importance of the nutrition area is sometimes forgotten and differs from hospital to hospital as far as the activities and cost objects are concerned.

This study also adds evidence that the ABC methodology can be implemented in a hospital. There are hospitals that are reluctant to use ABC because they believe that it is expensive and difficult to implement, that managers are not committed to such initiatives, and that the information is not fully leveraged in activity based systems [35]. However, this paper provides a replicable model for other nutrition departments that can be extended to the whole hospital.

ABC represents an opportunity to obtain a more detailed and rigorous method to allocate the costs for a nutrition unit. The results from this study yield a dollar amount per meal for a patient. This study, in fact, accomplished a series of objectives. First, ABC allows the identification of the processes and activities related with the food service of a hospital, which will help the management of the unit to understand their activities and resources. Second, there is a high level of information about the different meals produced in the hospital and their costs, which benefits the decision-making process of the services. This information was not available before. Third, there is a better understanding of what activities add value to the process of food production and delivery of the services. Fourth, it also allows one to determine the cost of activities, which are not directly related to nutritional activities, such as activities done for administrative purposes. Fifth, it provides useful information about the types and quantity of resources used and identifies possible sources of cost reduction, while maintaining the quality of services provided. Finally, a model for calculating indirect costs based on ABC can provide a stable basis for making managerial decisions in the nutrition unit and also meet the food standards that the Ministry of Health requires.

## Abbreviations

ABC: Activity based costing
ABM: Activity based management
